# Analysis of non-TIR NBS-LRR resistance gene analogs in *Musa acuminata *Colla: Isolation, RFLP marker development, and physical mapping

**DOI:** 10.1186/1471-2229-8-15

**Published:** 2008-01-30

**Authors:** Robert NG Miller, David J Bertioli, Franc C Baurens, Candice MR Santos, Paulo C Alves, Natalia F Martins, Roberto C Togawa, Manoel T Souza, Georgios J Pappas

**Affiliations:** 1 Postgraduate program in Genomic Science and Biotechnology, Universidade Católica de Brasília, SGAN 916, Módulo B, CEP 70.790-160, Brasília, DF, Brazil; 2 CIRAD/UMR DAP 1098, TA A 96/03 Avenue Agropolis, 34098 Montpellier Cedex 5, France; 3 EMBRAPA Recursos Genéticos e Biotecnologia, Parque Estação Biológica, CP 02372, CEP 70.770-900, Brasília, DF, Brazil

## Abstract

**Background:**

Many commercial banana varieties lack sources of resistance to pests and diseases, as a consequence of sterility and narrow genetic background. Fertile wild relatives, by contrast, possess greater variability and represent potential sources of disease resistance genes (R-genes). The largest known family of plant R-genes encode proteins with nucleotide-binding site (NBS) and C-terminal leucine-rich repeat (LRR) domains. Conserved motifs in such genes in diverse plant species offer a means for isolation of candidate genes in banana which may be involved in plant defence.

**Results:**

A computational strategy was developed for unbiased conserved motif discovery in NBS and LRR domains in R-genes and homologues in monocotyledonous plant species. Degenerate PCR primers targeting conserved motifs were tested on the wild cultivar *Musa acuminata *subsp. *burmannicoides*, var. Calcutta 4, which is resistant to a number of fungal pathogens and nematodes. One hundred and seventy four resistance gene analogs (RGAs) were amplified and assembled into 52 contiguous sequences. Motifs present were typical of the non-TIR NBS-LRR RGA subfamily. A phylogenetic analysis of deduced amino-acid sequences for 33 RGAs with contiguous open reading frames (ORFs), together with RGAs from *Arabidopsis thaliana *and *Oryza sativa*, grouped most *Musa *RGAs within monocotyledon-specific clades. RFLP-RGA markers were developed, with 12 displaying distinct polymorphisms in parentals and F1 progeny of a diploid *M. acuminata *mapping population. Eighty eight BAC clones were identified in *M. acuminata *Calcutta 4, *M. acuminata *Grande Naine, and *M. balbisiana *Pisang Klutuk Wulung BAC libraries when hybridized to two RGA probes. Multiple copy RGAs were common within BAC clones, potentially representing variation reservoirs for evolution of new R-gene specificities.

**Conclusion:**

This is the first large scale analysis of NBS-LRR RGAs in *M. acuminata *Calcutta 4. Contig sequences were deposited in GenBank and assigned numbers ER935972 – ER936023. RGA sequences and isolated BACs are a valuable resource for R-gene discovery, and in future applications will provide insight into the organization and evolution of NBS-LRR R-genes in the *Musa *A and B genome. The developed RFLP-RGA markers are applicable for genetic map development and marker assisted selection for defined traits such as pest and disease resistance.

## Background

Commercial banana varieties, which are mainly derived from *Musa acuminata *Colla, and *M. balbisiana *Colla, are cultivated in 130 countries across the tropics and sub-tropics, generating an annual production in excess of 100 million tons, and contributing significantly to food security [1]. Susceptible to over 50 fungal pathogens, as well as a number of bacterial pathogens, nematodes, viruses and insect pests, greatest threats to global banana production are currently caused by the fungal pathogens *Mycosphaerella fijiensis*, causal organism of black leaf streak disease (BLSD), and *Fusarium oxysporum *f. sp. *cubense *race 4, which causes Fusarium wilt. Agrochemical control of BLSD can be socio-economically and environmentally inappropriate, and requires integrated strategies to avoid the development of fungicide resistance in the pathogen. In the case of Fusarium wilt, however, chemical control is ineffective. For these reasons, the development of new disease resistant varieties is of paramount importance for the *Musa *industry. Although ranked as the fourth most important food commodity in terms of production value after rice, wheat and maize, genetic improvement of *Musa *has been limited. Cultivars have evolved from diploid, triploid and tetraploid wild species of *M. acuminata *(A genome) and *M. balbisiana *(B genome). Whilst wild species are generally fertile, many of today's commercial cultivars are sterile triploids or diploids, with fruit development via parthenocarpy. This translates to seedless fruits, or fruits which contain mostly non-viable seeds. As such cultivars have largely evolved via asexual vegetative propagation, their genetic base is narrow, with diversity dependent upon somatic mutation. Such limited genetic variation has resulted in a commercial crop that lacks resistance to pests and disease, as observed in cultivars such as Gros Michel and Grande Naine [2].

As sources of resistance to pathogens exist in germplasm, across the *Musa *genus, introgression of R-genes into susceptible cultivars offers potential for overcoming current constraints with conventional breeding. Resistant plant genotypes can prevent pathogen entry via a "gene for gene" defence mechanism, which, in the simplest model, is initiated through a direct or indirect interaction between a constitutive resistance (R) gene product and a specific biotrophic pathogen avirulence (Avr) gene product, or elicitor [3]. This recognition is postulated to trigger a chain of signal transduction events, leading to activation of defence mechanisms such as the hypersensitive response (HR), synthesis of antimicrobial proteins and metabolites, cell wall thickening and vessel blockage. Over the last 15 years, over 40 R-genes have been characterized from both model plants and important crop species [4], conferring resistance to several pathogens. Despite the wide range of recognized pathogen taxa, R-genes encode proteins that share significant sequence similarity and structural motifs, suggesting common protein-protein interactions as components of receptor systems and common roles in signalling events in plant defence responses.

To date, five principal classes of R-genes have been identified, based upon conserved protein domains (for review see [4]). The most abundant class are the cytoplasmic nucleotide-binding site-leucine-rich repeat (NBS-LRR) proteins [5]. The other classes comprise proteins with extracytoplasmic LRRs (eLRRs) anchored to a transmembrane (TM) domain (receptor-like proteins [RLPs]), cytoplasmic serine-threonine (Ser/Thr) receptor-like kinases (RLKs) with extracellular LRRs, cytoplasmic Ser/Thr kinases without LRRs, and proteins with a membrane anchor fused to a coiled coil (CC) domain. The common NBS-LRR-encoding proteins currently include over 20 functionally proven R-genes from diverse plant species [6,7]. Studies have focused on this family because its only known function to date is in disease resistance [8,9]. Gene products are composed of a conserved N-terminal NBS and variable length C-terminal LRR domain of 10 to 40 short LRR motifs [10]. The NBS domain is important for ATP binding and hydrolysis and is believed to be involved in signal transduction, triggered by the presence of the pathogen [11-13]. The LRR domain is likely to be involved in protein-protein interactions, recognizing pathogen elicitor molecules [14,15]. A high mutation rate in the LRR contributes to genetic variability, necessary for specific recognition of diverse pathogens [16]. Two subfamilies exist in NBS-LRR R proteins based upon N-terminal motifs. The TIR NBS subfamily R proteins display homology between the N-terminal amino acid motif and the receptor domain in *Drosophila Toll *and basal mammalian Interleukin (IL) 1 immunity factors in animals [17]. Non-TIR NBS subfamily R proteins can contain an N-terminal coiled-coil (CC) motif, a subset of which code for a leucine zipper sequence (LZ). TIR subfamily NBS-LRR proteins appear to be restricted to dicotyledons. As they have been reported in gymnosperms, grasses may have lost this type of R-gene family [18,19]. By contrast, non-TIR subfamily NBS-LRR proteins are present in both monocotyledons and dicotyledons [6]. Conserved amino acid motifs have been described in the NBS domains in these subfamilies [20], which include the phosphate-binding loop or 'P-loop' (also called kinase 1), kinase 2 [21,22], GLPL (also called kinase 3) and RNBS-A, B, C and D motifs [6]. The final amino acid within the kinase 2 motif can commonly reveal differences between TIR and non-TIR types, with an aspartic acid residue in TIRs and a tryptophan in non-TIRs [6].

Degenerate primers targeting conserved motifs have been used to amplify resistance gene analogs (RGAs) from diverse plant taxa such as soybean [23], *A. thaliana *[24], rice [25], and peanut [26], amongst others (for review see [27]). Many RGAs are phylogenetically related to known R-genes, and a number of studies have shown homologues mapping to R-gene loci (e.g. [23,24]), providing evidence that such genomic regions likely code for resistance. In *Musa*, progress in RGA characterization began recently, with only nine NBS-LRR disease resistance-like protein sequences currently deposited in GenBank (accessed December 2007). A number of non-TIR NBS RGAs have been amplified in wild *M. acuminata *and *M. balbisiana *accessions Gongjiao, Xinyiyejiao, as well as in cultivated species Zhongshandajiao, Fenjiao and Williams [28]. Other groups have described *Cf *orthologs in landrace Zebrina GF [29], and *Pto *family RGAs in *M. acuminata *cv Tuu Gia [30]. Characterization of NBS RGAs has also recently been extended to *Musa *species *M. ornata*, *M. schizocarpa*, *M. textilis*, and *M. velutina *[31].

Given that sequences so far studied are likely to represent only a small fraction of these resistance gene families in *Musa*, the objectives of this study were to identify NBS-LRR RGAs and explore their diversity in *M. acuminata *subsp. *burmannicoides*, var. Calcutta 4. This wild diploid cultivar has been used extensively in breeding programs, offering resistance to important fungal pathogens and nematodes. We describe a computational strategy for motif discovery, enabling PCR amplification of target motifs within NBS and LRR domains, and potentially applicable across different monocotyledonous species. Applied together with universal TIR and non-TIR NBS-targeting degenerate primers, we report the first large scale analysis of RGAs in *M. acuminata *Calcutta 4. Evolutionary relationships both among *Musa *sequences and RGAs from *A. thaliana *and *O. sativa *were determined, and polymorphic RFLP-RGA markers identified against *M. acuminata *mapping population parentals. Selected sequences were used to identify putative resistance gene loci across *M. acuminata *Calcutta 4, *M. acuminata *Grande Naine and *M. balbisiana *Pisang Klutuk Wulung BAC libraries.

## Results

### Degenerate primer design

**Figure 1:**
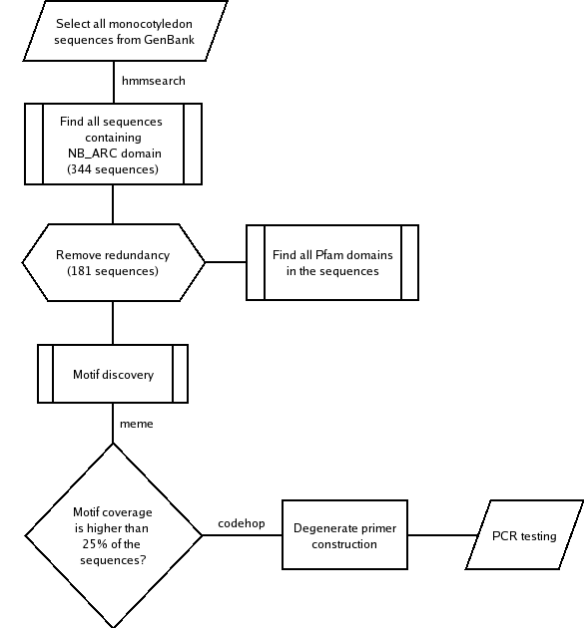
Computational protocol for primer design targeting motifs in non-TIR NBS and LRR domains in monocotyledons.

Public databases at present contain only very limited numbers of *Musa *R-gene or RGA sequences. In order to enrich the fraction of RGA candidates in *Musa *recoverable by PCR, an *in silico *protocol was devised to facilitate design of degenerate primers derived from monocotyledon sequences and targeting NBS and additional domains. Figure 1 depicts the process, beginning with HMMER-based selection of monocotyledon sequences from GenBank containing a characteristic domain shared by R-genes (Pfam id: NB-ARC). Following removal of redundant sequences (using a 95% identity threshold), 181 RGA candidates were obtained. Based on this subset, a search for conserved sequence motifs was conducted using the program MEME [32]. NBS-family motifs (P-loop, Kinase-2, GLPL, RNBS-D) were observed across the sequences, as well as novel conserved motifs outside the NBS domain, mostly within the LRR domain. All the conserved motifs identified served as candidates for degenerate primer design, with an additional constraint imposed, whereby motifs or close variants had to be present in at least 25% of the sequences (motif coverage). Primer design was conducted using the program CODEHOP [33].

### Isolation of NBS-LRR RGAs

A total of 860 high quality sequences were generated from insert-containing recombinant plasmids, of which 174 showed significant similarity to known *A. thaliana *R-genes and homologues (E-value ≤ 10^-5^), based upon searches using the BLASTX program. These sequences were obtained by PCR amplification with two distinct groups of primer combinations: universal primers taken from literature [23,26,34] and primers designed in this study. Universal TIR and non-TIR NBS-LRR-targeting primer combinations 1–7 (Table 1) resulted in PCR products of expected size, with P-loop to GLPL primer pairs yielding a single DNA band of approximately 650 bp, and P-loop to RNBS-D primer combinations a product close to 700 bp. High quality sequences were generated from 168 distinct clones, of which, following trimming and vector masking, 36 (21.43%) showed similarity with NBS-containing proteins in *A. thaliana*. The percentage of clones displaying similarity to RGAs varied between different primer combinations, ranging from 0–68% (Table 2).

**Table 1:** Degenerate primer sequences and target motifs used for RGA isolation in *M. acuminata *Calcutta 4

**Degenerate Primer**	**Target motif name/plant origin**	**Primer sequence (5' to 3')^a^**	**Author**
P1A (forward)	P-loop/Dicotyledon	GGIATGCCIGGIIIIGGIAARACIAC	[26]
P1B (forward)	P-loop/Dicotyledon	GGIATGGGIGGIIIIGGIAARACIAC	[26]
LM638 (forward)	P-loop/Monocotyledon & Dicotyledon	GGIGGIGTIGGIAAIACIAC	[23]
P3A (reverse)	GLPL/Dicotyledon	AIITYIRIIRYIAGIGGYAAICC	[26]
P3D (reverse)	GLPL/Dicotyledon	AIITYIRIIRYYAAIGGIAGICC	[26]
RNBSD-rev (reverse)	RNBS-D non-TIR/Monocotyledon & Dicotyledon	GGRAAIARISHRCARTAIVIRAARC	[34]
39F1 (forward)	Non NBS (n-terminal)/monocotyledon	TCATCAAGGACGAGCTGgarwbnatgma	This study
1F (forward)	P-loop – GKTT/monocotyledon	GGCGGGGTGGGCaaracnacnht	This study
P1C (forward)	P-loop – GKTT/Dicotyledon	GGICGICCIGGIIIIGGIAARACIAC	This study
3F2 (forward)	Kinase 2/monocotyledon	GAGGTACTTCCTGGTGCTGgaygayrtbtgg	This study
2F (forward)	RNBS-B/monocotyledon	AACGGCTGCAGGATCATGrtbachachmg	This study
1R1 (reverse)	P-loop/monocotyledon	CGTGCTGGGCCAGGgtngtyttncc	This study
P3B (reverse)	GLPL/Dicotyledon	AIITYIRIIRYIAGIGGIAGICC	This study
13R1 (reverse)	LRR/monocotyledon	CGGCCAAGTCGTGCAyvakrtcrtgca	This study
11R1 (reverse)	LRR/monocotyledon	TCAGCTTGCCGATCCACtydggsagbyt	This study

^a ^Degenerate code: I = inosine; R = A/G; Y = C/T; M = A/C; K = G/T; W = A/T; S = C/G; B = C/G/T; D = A/G/T; H = A/C/T; V = A/C/G; N = A/C/G/T

**Table 2:** *M. acuminata *Calcutta 4 amplicons obtained using degenerate RGA primers

**Primer Combinations**	**Target conserved motifs**	**Target Domains**	**Number of insert-containing plasmids producing high quality sequences**	**Number of sequences with homology to R-genes or RGAs^a^**
1. P1A-P3A	P-loop and GLPL	TIR and non-TIR NBS	28	8 (29%)
2. P1A-P3D	P-loop and GLPL	TIR and non-TIR NBS	33	1 (3%)
3. P1B-P3A	P-loop and GLPL	TIR and non-TIR NBS	36	4 (11%)
4. P1B-P3D	P-loop and GLPL	TIR and non-TIR NBS	19	1 (5%)
5. P1A-RNBSD-rev	P-loop and RNBS-D non-TIR	non-TIR NBS	9	1 (11%)
6. P1B-RNBSD-rev	P-loop and RNBS-D non-TIR	non-TIR NBS	31	21 (68%)
7. LM638-RNBSD-rev	P-loop and RNBS-D non-TIR	non-TIR NBS	12	0 (0%)
8. 39F1-1R1	Non NBS (n-terminal) and P-loop	NBS	no amplicon	na
9. 1F-P3B	P-loop and GLPL	non-TIR NBS	465	15 (3%)
10. P1C-P3B	P-loop and GLPL	NBS	no amplicon	na
11. 3F2-13R1	Kinase 2 and LRR	non-TIR NBS-LRR	227	123 (54%)
12. 3F2-11R1	Kinase 2 and LRR	NBS-LRR	no amplicon	na
13. 2F-13R1	RNBS-B and LRR	NBS-LRR	no amplicon	na
14. 2F-11R1	RNBS-B and LRR	NBS-LRR	no amplicon	na

^a^BLASTX analyses against a local database of *A. thaliana *R-genes and homologues utilized a minimum E-value of ≤ 10^-5^, na = not applicable

Primer combinations 8–14, which were derived from the computational pipeline described in Figure 1, targeted conserved amino acid motifs in non-TIR NBS-LRR sequences in monocotyledon and dicotyledon plants (Table 1). Combinations eight (39F1-1R1), 10 (P1c-P3b), 12 (3F2-11R1), 13 (2F-13R1), and 14 (2F-11R1) did not amplify reproducible PCR products. By contrast, primer combinations 11 (3F2-13R1) and nine (1F-P3b) consistently amplified products of approximately 650 bp in size, with 138 sequences showing similarity to RGAs in *A. thaliana*. Combination 11 was the more efficient of the two, with 54% of clones homologous to R-genes or RGAs (Table 2).

Most sequences that were not RGAs showed similarity to retroelements. These can constitute a large fraction of the plant genome [35] and many R-gene loci have been reported to contain interspersed transposable elements [36,37]. Considerable amplification of retroelements may also be expected because of their high copy number at the start of the reaction [38], which results in competition during PCR, even when primer match is poor.

### Analysis of assembled RGA sequences

Assembly of all 174 RGA sequences generated 62 contigs, with 52 complete sequences between primers following re-sequencing of selected clones. Thirty three contigs showed uninterrupted open reading frames (ORFs) encoding RGAs, with the remainder containing premature stop codons, and/or frameshifts. These latter sequences are likely derived from pseudogenes, PCR mutants or artefacts. Translation of complete *Musa *NBS-encoding sequences produced an equal number of non-redundant protein sequences. The average size of trimmed complete sequences (without RGA primers) was 610 bp, with an average 4.6 sequence coverage per consensus. Maximum and minimum sizes for these sequences were 1365 bp and 273 bp, respectively. The largest contig (MaRGA41) was isolated using P-loop and GLPL-targeting primers (primer combination 3). The GLPL motif sequence was the rare variant GSPL; and perhaps because of this, the GLPL-based primer did not bind to this site, but to a 3'-distal site, which may explain the larger and unexpected size of this product. Interestingly, the isolation of an anomalously large RGA for exactly the same reason was also observed in *Arachis *[26]. The TIR NBS class RGAs have been reported to be absent in monocotyledon genomes [19], and within this study all *Musa *RGAs conformed to the non-TIR NBS class, with a final tryptophan residue present in the kinase 2 motif.

### Phylogenetic analysis

**Figure 2:**
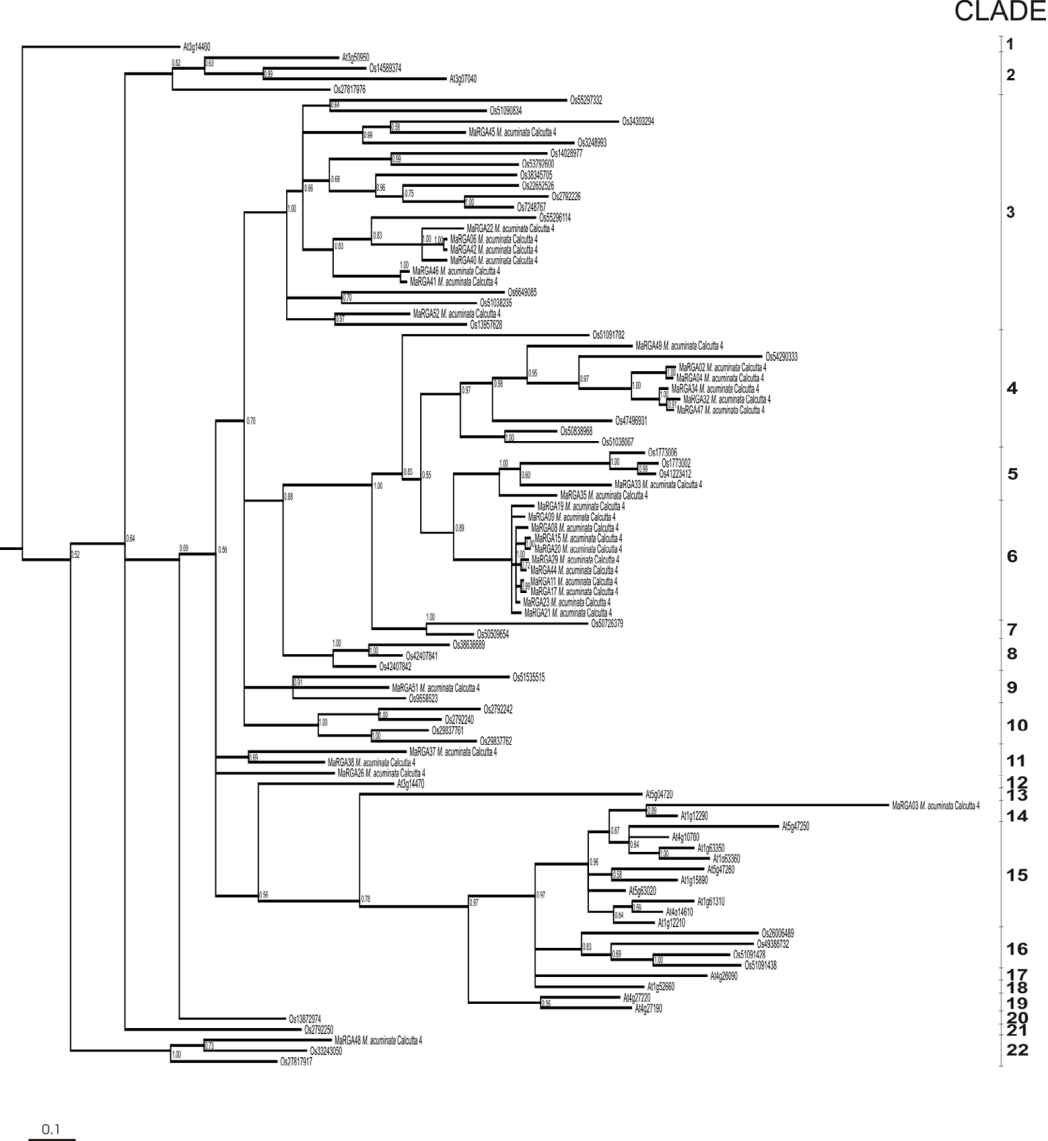
**Bayesian phylogenetic analysis of NBS-LRR amino acid sequences from *M. acuminata *Calcutta 4, *O. sativa *and *A. thaliana***. The majority rule consensus tree was derived from analysis of a common NBS region between the kinase 2 and GLPL motifs, and included 33 *M. acuminata *Calcutta 4 sequences, together with 21 representative non-TIR NBS-LRR domain sequences from *A. thaliana *and 43 from *O. sativa*. Clade numbers are included to facilitate discussion of data. All additional information for *Musa *tree sequences are summarised in Table 3. The branch lengths are proportional to the average number of amino acid substitutions per site, as indicated by the scale.

A Bayesian phylogenetic analysis of aligned amino acid sequences between the NBS kinase 2 and GLPL motifs was conducted in the 33 full length *Musa *sequences with contiguous ORFs, together with 21 representative non-TIR NBS-LRR class sequences from *A. thaliana *and 43 from *O. sativa *(Figure 2). Significant divergence was observed in the tree, with a total of 22 clades. Such variability has been described previously in non-TIR NBS sequences [10]. *Musa *sequences were divergent, indicating the presence of a diverse family of genes coding for proteins with NBS-LRR domains. Although dependent upon sample size, two clades contained sequences that appear to be specific to *M. acuminata *Calcutta 4 (clades 6 and 11). In contrast, a number of sequence types which may have expanded in monocotyledons were also observed, with *M. acuminata *Calcutta 4 sequences grouped together with a number from *O. sativa *(clades 3, 4, 5, 9 and 22). *Musa *RGAs also grouped with others from *A. thaliana *(clade 14), indicating amplification of conserved sequences which may be present throughout the angiosperms.

### RFLP-RGA markers

**Figure 3:**
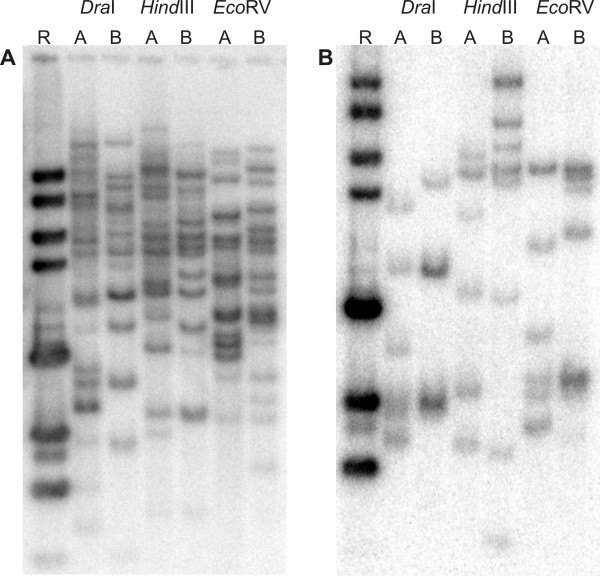
**Multiple loci polymorphisms observed in *M. acuminata *parentals with RGA genetic markers**. Polymorphisms were observed in *Dra*I, *Hind*III, and *Eco*RV-digested genomic DNA from *M. acuminata *spp. microcarpa genetic map parentals Borneo and Pisang Lilin, following hybridization of Southern blots with RGA probes MaRGA08 (panel A) and MaRGA37 (panel B).

**Figure 4:**
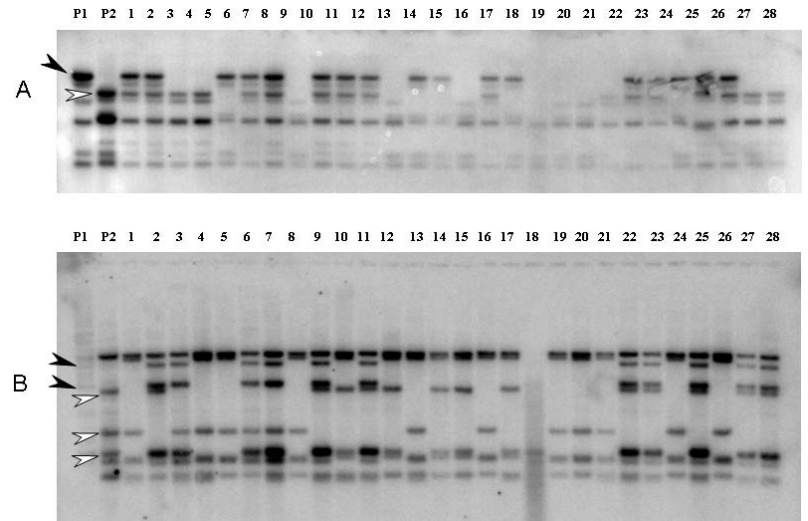
**Segregation of polymorphic bands in a subset of *M. acuminata *mapping population F1 progeny**. Hybrization of RGA probes MaRGA12 (Panel A) and MaRGA37 (Panel B) onto parentals and F1 progeny. P1: Pisang Lilin; P2: Borneo; and lanes 1 to 28: individual F1 plants. Segregating bands selected for mapping from P1 and P2 are indicated by black and white arrowheads, respectively.

From a total of 33 *Musa *RGAs evaluated as RFLP markers with restricted genomic DNA from mapping population parentals *M. acuminata *Borneo and Pisang Lilin, 30 displayed single locus or multiple loci polymorphisms on parentals, with at least one restriction enzyme (Table 3). Across the polymorphisms observed, 12 distinct fingerprint types were observed, when using enzymes *Dra*I and *Hind*III. RGA probes MaRGA04, MaRGA07, MaRGA08, MaRGA12, MaRGA13, MaRGA14, MaRGA16, MaRGA22, MaRGA37, MaRGA41, MaRGA43, and MaRGA46 represented each polymorphism pattern. Figure 3 shows examples of multiple loci polymorphisms observed on Southern blots of restricted parental DNA hybridized with probes MaRGA08 and MaRGA37. Segregation of selected polymorphic bands according to Mendelian ratios in a subset of F1 progeny for this mapping population is depicted in Figure 4.

**Table 3:** *Musa *RGA contig characteristics and polymorphic RFLP-RGA marker identification

**RGA contig^a^**	**Primer pairs**	**Size (bp)**	**Additional contig sequence information**	**Clade^b^**	**Polymorphisms observed on *M. acuminata *parentals^c^**		
					*Dra*I	*Hind*III	*Eco*RV

MaRGA01	1F-P3B	273	short, low homology	ni	nt	nt	nt
MaRGA02	1F-P3B	493	contiguous ORF	4	nt	nt	nt
MaRGA03	1F-P3B	481	contiguous ORF	14	Monomorphic	monomorphic	polymorphic (multiple loci)
MaRGA04*	1F-P3B	493	contiguous ORF	4	polymorphic (multiple loci)	polymorphic (multiple loci)	polymorphic (multiple loci)
MaRGA05	1F-P3B	316	contiguous ORF	ni	nt	nt	nt
MaRGA06	1F-P3B	647	contiguous ORF	3	nt	nt	nt
MaRGA07*	3F2-13R1	563	contiguous ORF	ni	polymorphic (multiple loci)	polymorphic (multiple loci)	polymorphic (multiple loci)
MaRGA08*	3F2-13R1	630	contiguous ORF	6	polymorphic (multiple loci)	polymorphic (multiple loci)	polymorphic (multiple loci)
MaRGA09	3F2-13R1	630	contiguous ORF	6	polymorphic (multiple loci)	polymorphic (multiple loci)	polymorphic (multiple loci)
MaRGA10	3F2-13R1	629	frameshift	ni	polymorphic (multiple loci)	polymorphic (multiple loci)	polymorphic (multiple loci)
MaRGA11	3F2-13R1	583	contiguous ORF	6	polymorphic (multiple loci)	polymorphic (multiple loci)	polymorphic (multiple loci)
MaRGA12*	3F2-13R1	531	frameshift, stop codon	ni	polymorphic (multiple loci)	polymorphic (multiple loci)	polymorphic (multiple loci)
MaRGA13*	3F2-13R1	587	contiguous ORF	ni	monomorphic	polymorphic (multiple loci)	monomorphic
MaRGA14*	3F2-13R1	501	contiguous ORF	ni	polymorphic (multiple loci)	polymorphic (multiple loci)	polymorphic (multiple loci)
MaRGA15	3F2-13R1	634	contiguous ORF	6	polymorphic (multiple loci)	polymorphic (multiple loci)	polymorphic (multiple loci)
MaRGA16*	3F2-13R1	454	contiguous ORF	ni	polymorphic (single locus)	monomorphic	monomorphic
MaRGA17	3F2-13R1	631	contiguous ORF	6	polymorphic (multiple loci)	polymorphic (multiple loci)	polymorphic (multiple loci)
MaRGA18	3F2-13R1	525	contiguous ORF	ni	monomorphic	polymorphic (multiple loci)	polymorphic (multiple loci)
MaRGA19	3F2-13R1	655	contiguous ORF	6	polymorphic (multiple loci)	polymorphic (multiple loci)	polymorphic (multiple loci)
MaRGA20	3F2-13R1	585	contiguous ORF	6	polymorphic (multiple loci)	polymorphic (multiple loci)	polymorphic (multiple loci)
MaRGA21	3F2-13R1	629	contiguous ORF	6	n/t	n/t	n/t
MaRGA22*	3F2-13R1	597	contiguous ORF	3	polymorphic (multiple loci)	polymorphic (multiple loci)	polymorphic (multiple loci)
MaRGA23	3F2-13R1	583	contiguous ORF	6	polymorphic (multiple loci)	polymorphic (multiple loci)	polymorphic (multiple loci)
MaRGA24	3F2-13R1	525	contiguous ORF	ni	monomorphic	polymorphic (multiple loci)	polymorphic (multiple loci)
MaRGA25	3F2-13R1	524	contiguous ORF	ni	monomorphic	polymorphic (multiple loci)	polymorphic (multiple loci)
MaRGA26	3F2-13R1	610	contiguous ORF	11	nt	nt	nt
MaRGA27	3F2-13R1	467	frameshifts, stop codons	ni	polymorphic (multiple loci)	polymorphic (multiple loci)	polymorphic (multiple loci)
MaRGA28	3F2-13R1	526	frameshift	ni	polymorphic (multiple loci)	polymorphic (multiple loci)	polymorphic (multiple loci)
MaRGA29	3F2-13R1	551	contiguous ORF	6	polymorphic (multiple loci)	polymorphic (multiple loci)	polymorphic (multiple loci)
MaRGA30	P1B-RNBS-D	675	translation unclear	ni	nt	nt	nt
MaRGA31	P1B-RNBS-D	1314	frameshift	ni	nt	nt	nt
MaRGA32	P1B-RNBS-D	633	contiguous ORF	4	nt	nt	nt
MaRGA33	P1B-RNBS-D	673	contiguous ORF	5	nt	nt	nt
MaRGA34	P1B-RNBS-D	792	contiguous ORF	4	nt	nt	nt
MaRGA35	P1B-RNBS-D	624	contiguous ORF	5	nt	nt	nt
MaRGA36	P1B-RNBS-D	675	contiguous ORF	ni	nt	nt	nt
MaRGA37*	P1B-P3D	472	contiguous ORF	11	polymorphic (multiple loci)	polymorphic (multiple loci)	polymorphic (multiple loci)
MaRGA38	P1A-P3A	472	contiguous ORF	11	monomorphic	monomorphic	monomorphic
MaRGA39	P1A-P3A	480	frameshift	ni	monomorphic	monomorphic	monomorphic
MaRGA40	P1A-P3A	860	contiguous ORF	3	polymorphic (multiple loci)	polymorphic (multiple loci)	polymorphic (multiple loci)
MaRGA41*	P1B-P3A	1365	contiguous ORF	3	polymorphic (multiple loci)	monomorphic	monomorphic
MaRGA42	3F2-13R1	619	contiguous ORF	3	No hybridization	No hybridization	No hybridization
MaRGA43*	1F-P3B	359	low homology	ni	polymorphic (multiple loci)	polymorphic (multiple loci)	polymorphic (multiple loci)
MaRGA44	3F2-13R1	631	contiguous ORF	6	polymorphic (multiple loci)	polymorphic (multiple loci)	polymorphic (multiple loci)
MaRGA45	3F2-13R1	625	contiguous ORF	3	polymorphic (multiple loci)	polymorphic (multiple loci)	polymorphic (multiple loci)
MaRGA46*	3F2-13R1	604	contiguous ORF	3	monomorphic	polymorphic (multiple loci)	monomorphic
MaRGA47	P1B-RNBS-D	636	contiguous ORF	4	nt	nt	nt
MaRGA48	P1B-RNBS-D	704	contiguous ORF	22	nt	nt	nt
MaRGA49	P1B-RNBS-D	1674	contiguous ORF	4	nt	nt	nt
MaRGA50	P1B-RNBS-D	633	frameshift	ni	nt	nt	nt
MaRGA51	P1A-RNBS-D	668	contiguous ORF	9	nt	nt	nt
MaRGA52	P1B-RNBS-D	669	contiguous ORF	3	nt	nt	nt

^a ^Contigs marked with asterisks were selected as polymorphic markers for inclusion on a *M. acuminata *genetic map

^b ^ni = not included in phylogenetic analysis

^c ^nt = not tested as genetic markers

### Physical distribution of *Musa *RGAs

**Figure 5:**
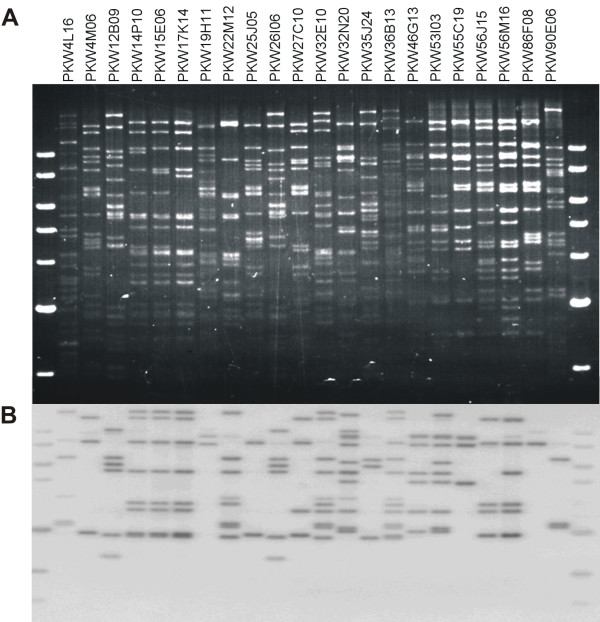
**RFLPs (A) and re-validated BAC clones (B) in *M. balbisiana*, identified with probe MaRGA08**. Panel A shows a fingerprinting gel of BAC clones digested with *Eco*RI, stained with ethidium bromide. Panel B shows results of hybridization of the Southern blot from panel A with radiolabelled probe MaRGA08.

*Musa *RGAs were used to screen BAC libraries derived from the wild type species *M. acuminata *Calcutta 4 (AA), *M. balbisiana *Pisang Klutuk Wulung (PKW) (BB) and the commercial triploid *M. acuminata *Grande Naine (AAA). In order to maximise identification of BAC clones containing target RGA loci, MaRGA08 and MaRGA37 were selected as probes, based upon differences in protein domains, motifs and phylogenetic clade. In all, 62 hits to BAC clones on high density filters were identified across the three genomes when screened with probe MaRGA08, and 43 hits when screened with probe MaRGA37. These clones were then fingerprinted and re-hybridized to their corresponding probe, to verify positive coordinates identified in the first screen and to provide data on copy number of NBS and NBS-LRR sequences across the three *Musa *genomes. A total of 88 out of 105 clones were verified, with only 17 clones failing to produce visible bands on Southern blots when hybridised to their respective probe (Table 4). False positives may have arisen as a result of identification of incorrect coordinates on BAC filters, failures in BAC plasmid preparation, problems in DNA blotting, or as a result of probe labelling or hybridization failure. MaRGA08 occurred as both a single copy and as multiple copies in validated BACs across the three genomes, with *M. acuminata *Calcutta 4 BAC clones harbouring mostly single-copy RGAs, in contrast to Grande Naine and PKW, where BACs contained up to nine and eleven copies, respectively. Figure 5 shows re-validated *M. balbisiana *BAC clones with high densities of this RGA. MaRGA37 was also present as multiple copies in validated BACs across the three genomes, with *M. acuminata *Calcutta 4 BAC clones harbouring up to six copies, PKW BAC clones two copies, and Grande Naine BACs containing up to nine copies. Both were therefore clearly members of multigene families, with a total of 232 copies of MaRGA08 and 183 copies of MaRGA37 observed in the positive clones identified across the 3 BAC libraries.

**Table 4:** Genomic organization of NBS-LRR loci across *M. acuminata and M. balbisiana *genomes

**RGA probe**	**BAC library**	**Number of positive BAC clones on high density BAC filters^a^**	**Number of positive BAC clones after Southern blot re-validation^b^**	**Validated BAC clone coordinates/number of copies of NBS-LRR sequences per BAC**
MaRGA08	*M. acuminata *Calcutta 4	16	11	52E23/7, 57G22/1, 68N02/1, 84K23/1, 105F04/1, 113F17/4, 114B14/1, 130I03/1, 137L16/1, 143P02/1, 97H24/1
MaRGA08	*M. acuminata *Grande Naine	24	18	24O03/1, 26P13/3, 62E05/4, 63A04/9, 66G14/3, 67F13/2, 75I23/3, 91O16/9, 95A22/6, 112K22/6, 114K14/9, 122D14/1, 125A08/4, 127O08/5, 133E15/6, 139M12/8, 142A24/3, 143P21/2
MaRGA08	*M. balbisiana *Pisang Klutuk Wulung	22	22	04L16/6, 04M06/3, 12B09/6, 14P10/7, 15E06/7, 17K14/7, 19H11/2, 22M12/8, 25J05/2, 26I06/6, 27C10/4, 32E10/11, 32N20/11, 35J24/3, 36B13/11, 46G13/6, 53I03/9, 55C19/3, 56J15/5, 56M16/7, 86F08/1, 90E06/3
MaRGA37	*M. acuminata *Calcutta 4	9	9	53G07/6, 56C23/6, 100K17/4, 100N08/4, 123I19/1, 126A11/1, 137L23/2, 139G23/1, 140M09/3
MaRGA37	*M. acuminata *Grande Naine	31	25	2A04/3, 17B04/7, 20I24/5, 24J20/6, 28K02/6, 32I11/7, 36G18/8, 47F09/4, 49B06/9, 49N21/7, 54B03/6, 59J09/7, 71F04/3, 73I23/7, 77K22/5, 79B08/7, 81P08/7, 88M19/6, 94D12/5, 94L13/8, 98K10/8, 106I23/6, 107M21/4, 121G06/4, 141M20/4
MaRGA37	*M. balbisiana *Pisang Klutuk Wulung	3	3	49E14/2, 86J02/2, 94I23/2

^a ^The BAC clones were identified following hybridization of probes MaRGA08 and MaRGA37 to *Musa *high density BAC filters

^b ^The BAC clones identified were reconfirmed via re-hybridization of probes MaRGA08 and MaRGA37 to Southern blots of restricted BAC clones

## Discussion

In contrast to most commercial *Musa *varieties, where genetic diversity is typically fixed by vegetative propagation, the sexually active cultivar *M. acuminata *Calcutta 4 represents an important source of novel genes for transfer across varieties. We report the first large scale analysis of NBS-LRR RGAs in this cultivar, using a degenerate primer design strategy devised for targeted RGA amplification across monocotyledon genomes. Given that R-genes are frequently located in clusters across genomes, with numerous copies of homologous sequences, *Musa *BACs containing RGAs were identified, as a resource for pinpointing candidate genes and for contributing to our understanding of R gene evolution. Polymorphic RGA genetic markers developed also offer potential for genetic improvement via marker assisted selection strategies.

### Characterization of RGAs

The PCR approach designed for RGA discovery in monocotyledon species was effective in *M. acuminata *Calcutta 4. All 174 cloned RGAs belonged to the non-TIR NBS-LRR subfamily, as expected, with considerable divergence observed at the amino acid level (Figure 2). From 52 complete NBS-encoding protein sequence contigs, 33 non-redundant sequences contained contiguous ORFs, which is a considerable number given that of the 157 putative genes in the *Arabidopsis *genome that code for NBS-type resistance proteins, 30% are of the non-TIR class [39]. However, our total may still reflect only a small portion of NBS-LRR sequences in *M. acuminata*, given that around 600 such sequences exist in rice [40]. All *Musa *RGAs encoded proteins with expected amino acid motifs, and showed homology to both putative R-genes and functional R-genes, such as At1g12290 in *A. thaliana*, which is a paralog of the R-gene *RPS5*, which confers resistance to *Pseudomonas syringae*. Of the *Musa *RGAs with contiguous ORFs, it is therefore possible that some may serve as functional R-gene candidates against diverse pathogens. Numerous pseudogenes were also co-amplified. These likely arise through point mutations, insertions or nucleotide deletions, acting as reservoirs for variation and offering the potential for recombination or gene conversion between R-gene alleles or paralogs [16]. In total, seven primer sets amplified RGAs, three targeting both universal TIR and non-TIR NBS motifs (primer pairs 1, 3 and 4), and four targeting non-TIR NBS motifs (primer pairs 5, 6, 9 and 11). A number of factors may have contributed to the success rate of primers. Our design strategy for monocotyledons took into account the number of degeneracies, primer length, nucleotide composition, degeneracy position within each primer, and prevalence of putative targets in the sequences analysed. Universal primer combinations designed for both TIR and non-TIR NBS motifs in dicot sequences were relatively inefficient, with a maximum of 29% of sequences homologous to RGAs when amplified with primer combination 1. Amplification was most efficient using non-TIR targeting primers, with 67.74% and 54.18% of sequences that were amplified, respectively, with primer combinations 6 and 11, showing significant similarity to R-genes and RGAs.

Phylogenetic analysis revealed considerable polymorphism, with *Musa *RGAs separating into eight distinct clades, with a number defining *Musa *specific clades. Such variability might be expected, given that non-TIR NBS-LRR sequences are often more heterogeneous than the TIR subfamily in plant taxa [10]. Sequences generated with primers targeting non-TIR motifs were more diverse than those produced with universal primers targeting motifs common to both TIR and non-TIR subfamilies. A higher degree of polymorphism exists in LRR domains in NBS-LRR family R-genes and homologues, as a result of diversifying selective pressure [16]. Primers targeting this domain are thus likely to promote amplification of diverse RGAs. Primer pair 11, the only to target both NBS and LRR motifs, was not only the second most efficient primer combination for RGA amplification, but also a primer pair amplifying diverse RGAs, which were spread across a number of clades. The literature shows that the NBS domain is present in both plant resistance genes, together with genes coding for kinases or ATP/GTP-dependent enzymes. By contrast, proteins containing both NBS and LRR domains have only been described in plant resistance genes so far. Given that primer combination 11 produced amplicons from the NBS kinase 2 to a conserved motif within the LRR domain, efficiency in amplification of targets involved in disease resistance is therefore potentially greater.

Diversity observed among the *Musa *RGAs suggests a contribution towards evolutionary fitness in the plant. Both R-genes and pathogen Avr genes are under constant evolutionary pressure, with mutation in the pathogen resulting in loss of resistance in the plant. Understanding R-gene evolution mechanisms is essential for determining how plants maintain their resistance to pathogens [21,41]. Potential genetic mechanisms responsible for R-gene genetic variation and evolution in plant taxa include recombination, gene conversion, unequal crossing over, transposable elements and point mutations, with the latter considered the principal evolutionary mechanism [16]. In general, sequence similarity was high between *Musa *sequences within each individual clade, suggesting recent evolutionary divergence. However, given that *Musa*-containing clades contained relatively few RGA contig sequences, tree topologies may only be approximate, as a result of insufficient sampling. As we targeted motifs present in at least 25% of monocotyledon-derived sequences containing the NBS-LRR domains, we are perhaps also biased to such sequences. A fully comprehensive analysis of non-TIR NBS-LRR sequences in *M. acuminata *will require multiple primer sets, together with more exhaustive sequencing of amplicons. Although our study did not report amplification of any TIR NBS-LRR RGAs, in agreement with the hypothesis that the TIR subfamily is restricted to dicotyledonous taxa [41], existence of the TIR motif has now been reported in the rice genome, albeit in reduced numbers [18,19]. Lack of detection in the *Musa *monocotyledon genome may therefore reflect limitations in PCR amplification.

### RGA applications in mapping

In support of the hypothesis that genes conferring quantitative resistance may show homology to R-genes, as originally proposed by [42], numerous RGAs have been mapped to genomic regions for quantitative trait loci associated with resistance (e.g. [23,43]). Within our study, RGAs displayed single locus or multiple loci polymorphisms on *M. acuminata *parentals. Similar degrees of polymorphism using RGAs as RFLP probes have been observed in rice [43]. Together with SSR and DArT markers, our RGAs have been included on a reference genetic map which is under development. As most mapping programs in *Musa *have faced problems with production and maintenance of large populations, mainly as a result of translocation events which complicate gamete formation and segregation [44,45], this latest attempt involves a cross between *M. acuminata *spp. microcarpa "Borneo" and *Musa acuminata *spp. malaccensis "Pisang Lilin", which is reported to carry only a single translocation event. This mapping project will serve as a base for development of a core set of markers for uptake in future mapping projects in banana. Analysis of our RGA markers on mapping populations segregating for resistance to biotic stresses is required to determine linkage between RGAs and R-gene loci. Such R-gene markers would be valuable in marker-assisted selection programs for trait selection. Utilized in high resolution genetic mapping, RGA markers may also serve as an effective approach for map-based cloning of *Musa *R-genes.

### Physical distribution of *Musa *RGAs

Clustering of multi-copy R gene families and RGAs is common in plant genomes [39,43] with up to 60% of R-genes clustered [46], as a result of tandem duplications of paralogous sequences [47]. As RGAs frequently cluster around such loci, they can therefore serve as useful candidates for R-gene discovery across BAC libraries. Eighty eight RGA-positive clones were revalidated, a number which is expected for R-genes, given that they are often members of large gene families. No co-hybridization was observed with probes MaRGA08 and MaRGA37. This is also perhaps expected, as probe sequences were phylogenetically distinct, and were amplified using primer sets targeting different motifs. Given that greater polymorphism is expected in LRR domains in NBS-LRR R-genes, comparison of number of BAC hits between the two RGA probes supported this idea. MaRGA08 was amplified with a primer pair targeting degenerate kinase 2 and LRR motifs, and the probe hybridized to a greater number of clones than MaRGA37, which targeted more conserved NBS P-loop and GLPL motifs. Analysis of copy number of RGAs in re-validated BAC clones (Table 4 and Figure 5) showed that in addition to occupying potential multiple loci across the three genomes, multiple copies are also common in positive BACs for both RGA probes. Probe MaRGA08, which targeted NBS-LRR sequences, revealed in general more copies per BAC than probe MaRGA37, which targeted NBS domains only. Given the greater diversity in LRR motifs, perhaps diversifying selection has resulted in an increase in NBS-LRR RGA copy number, via gene duplication. Within such RGA clusters, numerous R-genes may be present conferring resistance to different strains of a particular pathogen or to different pathogen taxa [48]. Such genomic organization may also represent a variation reservoir, from which new R-gene specificities may evolve.

Given that 33 contiguous *Musa *RGAs were identified in our study, further testing of additional probes representative of distinct clades against the *Musa *BAC libraries would likely identify more putative resistance loci across the three genomes. Ongoing shotgun sequencing of a number of clones from each of the *Musa *BAC libraries will contribute to our understanding of the organization and mechanisms governing evolution of NBS-LRR resistance gene regions, with comparisons of alleles within each genome and orthologs across the three genomes, and will provide an additional basis for genetic marker development.

## Conclusion

Given the low genetic diversity existent in commercial *Musa *cultivars, the rapid spread of fungal pathogens, together with the slow progress in gene discovery in *Musa*, this conserved orthologous sequences (COS) marker approach towards R-gene discovery was conducted, to provide potential opportunities for genetic improvement via marker assisted selection, genetic breeding and genetic engineering. This work, as well as reporting the first large scale analysis of RGA diversity in *M. acuminata *Calcutta 4, described a primer design strategy for NBS-LRR RGAs across monocotyledonous genomes, and developed RFLP-RGA markers for genetic mapping. RGA-containing BAC clones will serve as a resource for map-based cloning, and will contribute to our understanding of the organisation and evolution of NBS-LRR R-genes in the *Musa *A and B genomes.

## Methods

### Plant material and DNA extraction

*M. acuminata *Calcutta 4 plants [*Musa *Germplasm Information System (MGIS) accession number NEU0017, genus section EUMUSA] [49] were propagated *in vitro *and plants maintained in a greenhouse. Genomic DNA was extracted using a standard CTAB approach, with young leaf tissues macerated using a Bio 101 Thermo Savant FastPrep^® ^FP 120 cell disrupter (Qbiogene, Irvine, CA, USA). For RFLP-RGA marker development, genomic DNA was extracted from young leaves in *M. acuminata *mapping population parentals Borneo (NEU 0028 – ITC 0253) and Pisang Lilin (NEU 0063 – ITC001), together with F1 progeny, using a modified mixed alkyl trimethyl ammonium bromide (MATAB) procedure [50].

### Degenerate primer sets

**Figure 6:**
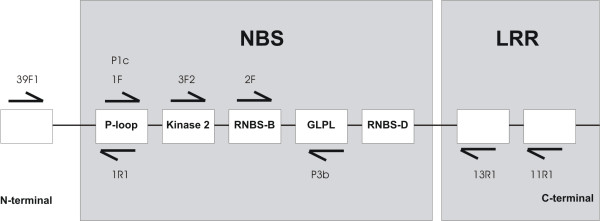
**Conserved motifs in non-TIR NBS and LRR domains targeted using degenerate RGA primers**. The arrows show primer positions, with tips indicating the 3' primer end. The scheme is not to scale.

Nine degenerate primers were designed in this study (Figure 6, Table 1). Seven were designed based upon conserved motifs in non-TIR NBS-LRR domain-containing monocotyledon sequences obtained from Genbank (primers 39F1, 1F, 3F2, and 2F [all forward], and 1R1, 13R1, and 11R1 [all reverse]). Primers P1C and P3B targeted non-TIR NBS-LRR protein motifs in dicotyledons, designed following alignment of resistance proteins tomato PRF (gi:1513144), tomato I2C-1 (gi:2258315), *A. thaliana *RPS2 (gi:548086) and RPS1 (gi:963017), and the *C. elegans *Cell death protein 4. Additional universal primers targeting both TIR and non-TIR NBS-LRR R proteins were also tested, which were previously designed from conserved motifs (Table 1) present in several dicotyledonous plant RGAs (*A. thaliana, Linum usitatissimum*, *Solanum lycopersicon*, *Nicotiana glutinosa *and *Solanum tuberosum*). Universal forward primers comprised P1A, P1B [26], and LM638 [23], and reverse primers P3A, P3D [26], and RNBSD-rev [34]. In total, 14 out of the 16 possible primer combinations were tested (Table 2).

### PCR amplification

Each PCR reaction was performed in a 25 μl volume, containing 50 ng of template genomic DNA, 2.5 mM MgCl_2_, 0.2 mM dNTPs, 0.5 μM of each primer, 1.25 U of Taq polymerase (Phoneutria, Belo Horizonte, MG, Brazil), and 1× IB Taq polymerase buffer (Phoneutria, Belo Horizonte, MG, Brazil). Temperature cycling was conducted with the following program: 96°C for 5 min; 35 cycles of 96°C for 1 min, 45°C for 1 min, and 72°C for 1 min; plus an extra elongation period of 10 min at 72°C.

### Cloning and sequencing

Following electrophoresis, PCR products of expected size were purified using a Qiagen QIAquick PCR purification kit (Qiagen, Valencia, Ca, USA). Products were cloned using either pGEM-T-Easy (Promega, Madison, WI, USA) or pCR2.1TOPO (Invitrogen, Carlsbad, CA, USA). Ligations were desalted using Millipore dialysis membranes (0.02 μM) and DH5α *Escherichia coli *cells were transformed either by electroporation using a GenePulser II (Bio-Rad, Hercules, CA, USA) set at 2.5 kV and 200Ω, or via a standard heat shock protocol. Recombinant plasmid clones were selected and manipulated following standard protocols [51]. Forward and reverse sequencing of clones was conducted on ABI 377 and 3700 DNA sequencers (Applied Biosystems, Foster City, CA, USA), using, for each respective sequencer, a DYEnamic ET Terminator Cycle Sequencing Kit (Amersham Biosciences, Piscataway, NJ, USA) and an ABI BigDye Terminator Cycle Sequencing Kit (Applied Biosystems, Foster City, CA, USA).

### Sequence analysis

Sequences were processed to remove vector and poor quality sequences using the Staden sequence analysis software package [52]. Contig assembly was performed using CAP3 [53] and by manual editing. RGAs were identified on the basis of sequence similarity using the program BLASTX [54], against a local database of *A. thaliana *R-genes and homologues, as described in [26]. Further confirmation was obtained by checking for the Pfam NB-ARC domain [12], which is a protein domain characteristic of plant resistance genes, using the program HMMER [55]. Only unbroken reading frames between the NBS domain kinase 2 and GLPL motifs (a common sequence to all generated contigs) were retained. Derived protein sequence alignments were obtained using the program MUSCLE [56], and included 21 representative non-TIR NBS-LRR sequences from *A. thaliana *and 43 from *O. sativa*. Bayesian phylogenetic inference was performed using the program MrBayes v.3.1.2 [57], according to the Jones+Gamma model, using 6 parallel Monte Carlo chains over 10^6 ^generations. The reliability of tree topologies was tested by bootstrapping 1000 times, with construction of a final majority rule consensus tree.

### Identification of polymorphic RGA genetic markers

*Musa *RGAs were hybridized on restricted genomic DNA of parentals *M. acuminata *spp. microcarpa Borneo and Pisang Lilin. Restriction enzyme survey test blots were conducted to identify probe/enzyme combinations revealing polymorphisms, using *Musa *RGA clones representative of 33 contigs as candidate RFLP probes. Parental genomic DNA (91 μg) was digested separately with 1040 U each of enzymes *Dra*I, *Eco*RV, and *Hind*III, followed by removal of proteins and salts. RFLP digests (20 μl) were separated by electrophoresis on 1% agarose gels run overnight at 30 V in 1× TAE buffer, together with 1 kb Ladder (Invitrogen, Carlsbad, CA, USA) and Raoul markers (Appligene, Illkirch, France). Southern blotting onto Hybond N+ membranes (Amersham Biosciences, Piscataway, NJ, USA) was conducted via capillary transfer with 0.25N HCl depurination solution, 0.4N NaOH neutralization solution, and 0.4N NaOH transfer solution, according to standard protocols. Test blot membranes were placed in pre-hybridization buffer (20 × SSPE, 20% SDS, Denhart's 50 ×, *E. coli *tRNA (10 mg/ml)) and incubated overnight at 65°C in a rotisserie oven. RGA probes were denatured at 95°C for 5 min, and 5 μl labelled with 4 μl (α-^32^P dCTP) via random hexanucleotide primed DNA synthesis using a Megaprime™ DNA Labelling System RPN 1607 (Amersham Biosciences, Piscataway, NJ, USA). Probes were added to 20 ml of hybridization solution (20 × SSPE, 20% SDS, Denhart's 50 ×, *E. coli *tRNA [10 mg/ml], 50% Dextran/H_2_O) and again incubated overnight at 65°C. In order to remove non-specific background following hybridization, membranes were washed at 65°C for 20 min, twice in wash solution 1 (5 × SSPE), once in wash solution 2 (1 × SSPE and 0.1 × SDS), and once in wash solution 3 (0.1 × SSPE and 0.1 × SDS). Membranes were air dried and hybridization fingerprints observed after both overnight exposure on a filmless autoradiography Storm 820 imaging system (Amersham Biosciences, Piscataway, NJ, USA) and a 7 day room temperature exposure using autoradiography film and intensifying screens. Scorable fragment length polymorphisms were examined for each RGA probe/restriction enzyme combination.

### Physical distribution of *Musa *RGAs

Two RGA probes were hybridized to high density colony filters representing previously constructed BAC libraries *M. acuminata *Calcutta 4 (AA) [58], *M. acuminata *Grande Naine (AAA) [59], and *M. balbisiana *Pisang Klutuk Wulung (PKW) (BB) [60]. Probe labelling, hybridization, washing and exposure procedures were as described earlier. For all positive BAC clones identified, 3 μl of glycerol stock for each clone were inoculated into 3 ml of 2 × YT medium containing chloramphenicol (12.5 μg/ml), and grown at 37°C for 14 h. BAC DNA isolation was conducted using a standard alkaline lysis procedure on a QIAGEN BIO ROBOT 9600 (Qiagen, Valencia, CA, USA). DNA samples (500 ng) were digested overnight with 40 U of *Hind*III or *Eco*RI (New England BioLabs, Ipswich, MA, USA), loaded onto 1% gels and fragments separated by overnight electrophoresis at 40 V. BAC clones initially identified on high density filters were validated by re-hybridization with the corresponding RGA probe used in initial BAC filter screening, using blotting and probe hybridization procedures as described for RGA genetic marker studies.

## Authors' contributions

All authors read and approved the final manuscript. RNGM participated in conceiving the study, DNA extraction, PCR amplification, library construction, genetic and physical mapping, and drafting the final manuscript. DJB participated in conceiving the study, primer design, and sequence analysis. FCB supervised mapping of RGA markers and physical mapping. CMRS, NFM and RCT participated in bioinformatics. PCA participated in PCR amplification and library construction. MTSJ participated in conceiving the study, DNA extraction, PCR amplification, and RGA library construction. GJPJ participated in conceiving the study, primer design strategy, and bioinformatics.
